# Function-specific virtual screening for GPCR ligands using a combined scoring method

**DOI:** 10.1038/srep28288

**Published:** 2016-06-24

**Authors:** Albert J. Kooistra, Henry F. Vischer, Daniel McNaught-Flores, Rob Leurs, Iwan J. P. de Esch, Chris de Graaf

**Affiliations:** 1 Amsterdam Institute for Molecules, Medicines and Systems (AIMMS), Division of Medicinal Chemistry, Faculty of Sciences, Vrije Universiteit Amsterdam, De Boelelaan 1108, 1081 HZ Amsterdam, The Netherlands

## Abstract

The ability of scoring functions to correctly select and rank docking poses of small molecules in protein binding sites is highly target dependent, which presents a challenge for structure-based drug discovery. Here we describe a virtual screening method that combines an energy-based docking scoring function with a molecular interaction fingerprint (IFP) to identify new ligands based on G protein-coupled receptor (GPCR) crystal structures. The consensus scoring method is prospectively evaluated by: 1) the discovery of chemically novel, fragment-like, high affinity histamine H_1_ receptor (H_1_R) antagonists/inverse agonists, 2) the selective structure-based identification of ß_2_-adrenoceptor (ß_2_R) agonists, and 3) the experimental validation and comparison of the combined and individual scoring approaches. Systematic retrospective virtual screening simulations allowed the definition of scoring cut-offs for the identification of H_1_R and ß_2_R ligands and the selection of an optimal ß-adrenoceptor crystal structure for the discrimination between ß_2_R agonists and antagonists. The consensus approach resulted in the experimental validation of 53% of the ß_2_R and 73% of the H_1_R virtual screening hits with up to nanomolar affinities and potencies. The selective identification of ß_2_R agonists shows the possibilities of structure-based prediction of GPCR ligand function by integrating protein-ligand binding mode information.

In the past decade, there has been remarkable progress in the structural elucidation of G protein-coupled receptors (GPCRs), the largest family of transmembrane proteins in the human genome that plays an essential role in a plethora of cell signalling processes and has high potential as drug targets^1^,^2^. Currently, over 140 GPCR crystal structures have been published, covering 33 different GPCRs and 72 unique small molecule GPCR ligands with different functional effects on receptor signalling and with distinct binding modes in the receptor^1^,^2^. The increased amount of high resolution structural information on GPCRs has opened up new opportunities for the identification of novel GPCR ligands by structure-based virtual screening (SBVS)^3^,^4^,^5^,^6^. There are, however, still several hurdles for structure-based approaches for GPCRs, such as the efficient identification of chemically novel fragment-like ligands with high hit rates^4^,^7^ (i.e. the portion of experimentally validated hits) and the structure-based prediction of GPCR ligand function^5^,^6^,^8^. The last-mentioned hurdle has proven to be challenging as the functional effect of a ligand is inherently linked to the binding mode(s) it can adopt, and the receptor conformations it can stabilize that are associated with specific receptor activation states^9^,^10^. The development of structure-based, ligand-function specific virtual screening methods is hampered by the fact that for most crystallized GPCRs only one or few structures are available as well as the limited diversity of the functional effect and binding modes of the co-crystallized ligands. Whereas the efficient identification of fragment-like compounds was shown to be hampered primarily by the (target-dependent) inability of docking scoring functions to accurately rank and score the different binding modes with an estimation of their binding energies^11^. These challenges are, for example, illustrated by a recent structure-based virtual screening study by Rodríguez *et al*. in which high-affinity adenosine antagonists were discovered while screening against an active-state agonist-bound X-rays of the A_2A_ receptor in order to obtain A_2A_ agonists^12^. In another virtual screening study against the ß_2_-adrenoceptor (ß_2_R), Weiss *et al*. were able to identify 1 fragment-like and 5 lead-like ß_2_-adrenoceptor (ß_2_R) agonists by making use of both an active-state agonist-bound and an inactive-state inverse-agonist bound structure and selecting compounds that were only scored high in the agonist structure^13^. In order to overcome issues with scoring and ranking docking poses consensus approaches have been devised^14^. These consensus scoring approaches have been applied retrospectively^14^,^15^ and prospectively^16^,^17^,^18^ in several studies. In those cases where the consensus approach has been prospectively applied the individual approaches have, however, never been experimentally validated. Only the prospective application, experimental validation and comparison of the individual and combined scoring methods would allow the assessment of the added value of consensus scoring in virtual screening.

In the current study we address both hurdles in virtual screening simultaneously by applying a novel docking scoring approach for the identification of novel fragment-like GPCR ligands and the prediction of their functional effect using GPCR crystal structures. This docking scoring approach combines a conventional docking scoring function (ChemPLP) using PLANTS^19^ docking with the molecular interaction fingerprint (IFP) rescoring approach^20^,^21^. This combination is based on the “complementary” hypothesis in which it is assumed that the combination of two (fundamentally) different scoring functions can result in increased performance by combining the strengths of each scoring function^14^. ChemPLP is an empirical energy-based scoring function whereas IFP compares the interaction pattern between the docking pose and the protein to a reference binding mode, most often the co-crystallized pose of a known ligand. By combining these methods we aimed to use the strengths of PLANTS to identify compounds with energetically favorable docking poses with the strength of IFP to select the most probable binding modes by selecting those with an interaction profile closely resembling the reference IFP^20^ (in this case the IFP of the co-crystallized ligand with the receptor). Moreover, in this study we have validated the performance of the consensus scoring approach in a truly prospective manner by also experimentally validating the results of the individual scoring approaches. We applied and systematically compared the combined and individual IFP and PLANTS scoring approaches with respect to their ability to: 1) identify chemically novel, fragment-like, high affinity histamine H_1_ receptor (H_1_R) antagonists/inverse agonists, and 2) selectively retrieve ß_2_R agonists. The ß_2_R represents a rewarding additional target as there are many crystal structures available and we aim to selectively identify agonists, thereby representing a complementary case study to H_1_R. Building from our successful prospective H_1_R VS study^21^ and retrospective ß_2_R VS study^22^ reported earlier, we have in the current study for the first time explored and compared the virtual screening performances of the different scoring approaches and combinations for H_1_R and ß_2_R in a prospective manner.

The integration of protein-ligand interaction energy and interaction pattern similarity scores resulted in a better performance than each of the individual methods, although in all cases high hit-rates were obtained. Retrospective virtual screening studies based on multiple different ß-adrenoceptor crystal structures allowed us to select an optimal combination of reference interaction fingerprint and protein conformation for the selective retrieval of novel, fragment-like ß_2_R *agonists*. These results demonstrate the potential of structure-based prediction of GPCR ligand function by the integration of protein-ligand binding mode information.

## Results and Discussion

### Discovery new fragment-like H_1_R ligands

**Figure 1:**
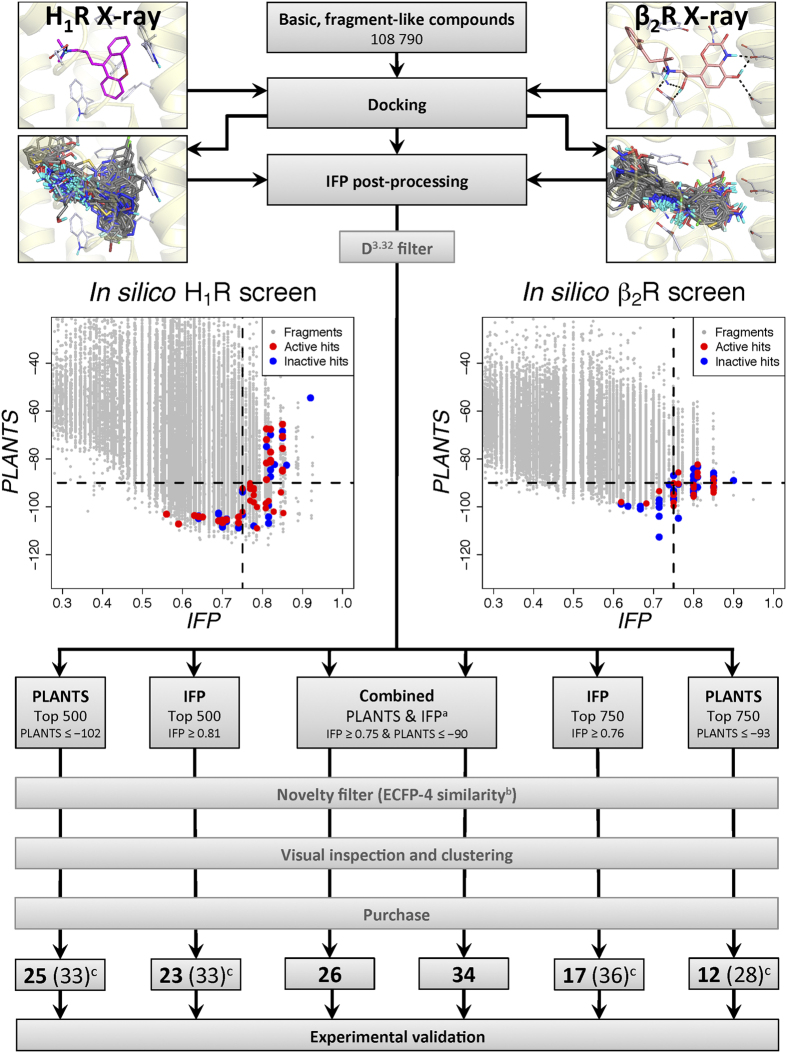
Workflow of the virtual-screening approaches performed on both the H_1_R and the β_2_R. The indicated PLANTS and IFP cut-offs for the top 500/750 compounds are indicative, only compounds within the top 500/750 compounds were selected for further processing. Notes: (**a**) The definition of these cut-offs has been described. (**b**) An ECFP-4 similarity cut-off of 0.4 and 0.5 was used for the H_1_R and ß_2_R selections, respectively. (**c**) The number before the brackets indicate the number of compounds that are unique to this selection, the number between brackets include compounds from the combined selection that match the criteria of the individual approach (see Fig. 3).

**Figure 2:**
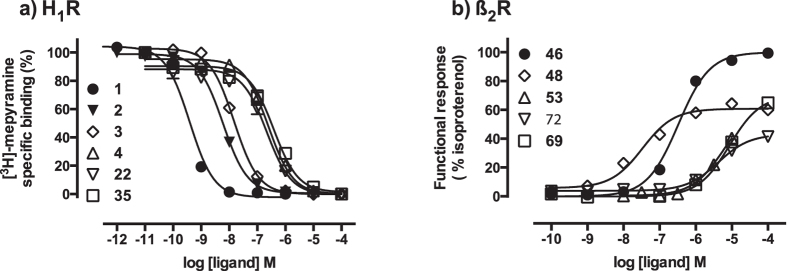
Representative radioligand displacement (H_1_R, reference compounds **1** and **2**) and functional-response (ß_2_R, reference compound **46**) curves of the reference ligands and selected compounds identified using the structure-based virtual screening on H_1_R (**a**) and ß_2_R (**b**). Curves for selected compounds from each of the scoring approaches curves are presented in Supplementary Fig. S2.

In order to analyse to what extent the combined scoring approach was responsible for the high hit-rate of our previously reported virtual screening on the doxepin-bound (**1**) H_1_R crystal structure (PDB-code 3RZE^23^) we experimentally validated the compound selections for each of the individual scoring approaches (Fig. 1). For both the PLANTS as well as the IFP approach the top 500 scoring compounds were selected and subsequently the compounds matching the combined approach were removed. The remaining compounds were processed in the same fashion as the compounds from the combined approach. We excluded hits that are similar to any known H_1_R ligand (ECFP-4^24^ Tanimoto score of ≥0.4), visually clustered the compounds based on scaffold similarity, and discarded compounds with buried polar groups that were placed in hydrophobic parts of the H_1_R binding site by visual inspection. In total 74 compounds were purchased and tested for their H_1_R affinity (including the 26 compounds reported in the previous article^21^). Table 1 gives an overview of the results and Fig. 2a illustrates the robustness of the assays performed.

### Discovery new β_2_R agonists

**Figure 3:**
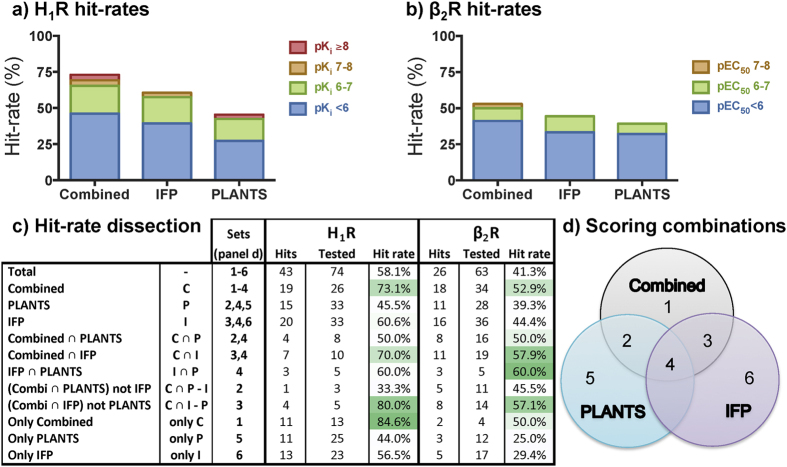
Hit rate analysis for each of the scoring approaches. Bar-plots summarizing the hit-rates and the affinity/potency of the experimentally validated hits for both (**a**) the H_1_R and (**b**) the β_2_R virtual screening. (**c**) Analysis of the hit-rates for each of the scoring methods and all possible combinations thereof (as highlighted in panel (**d**)) for both H_1_R and β_2_R. Hit rates for each set (panel (**d**)) are reported as the number of hits and the total number of tested compounds followed by the hit rate percentage. (**d**) Venn diagram highlighting the different scoring combinations used for the analysis of H_1_R and β_2_R hit-rates in panel (**c**).

For the selection of the optimal β-adrenoceptor crystal structure we performed a retrospective virtual screening study (in line with the study performed by De Graaf *et al*.^25^) on 6 β_1_R and 7 β_2_R ligand-bound crystal structures (PDB accessed May 2011)^26^,^27^,^28^,^29^,^30^,^31^,^32^,^33^,^34^. β_1_R structures were also taken into account because of the high ligand and binding site similarity with β_2_R^35^. This retrospective VS analysis highlighted that the (first) active-state β_2_R crystal structure with the G_s_-mimicking nanobody and the full agonist BI-167107 (**47)** (PDB-code 3P0G, Figs 1 and 3) was found to have one of the highest retrieval rate for agonists (EF_1%_ 76.9) while maintaining a low retrieval rate for antagonists/inverse agonists (EF_1%_ 7.7) when using the IFP scoring approach (see Supplementary Fig. S1). It should be noted that meanwhile, 35 β-adrenoceptor crystal structures have been deposited in the PDB (see Table S1). Systematic retrospective virtual screening studies against 31 of the β-adrenoceptor crystal structures indicated that docking and IFP scoring in 3P0G still gives one of the highest and most selective enrichments for the discrimination of partial/full agonists versus decoy molecules and inverse agonists/antagonists^22^. We performed a virtual screening on the selected active-state β_2_R structure using the same approach and fragment library as used for the H_1_R virtual screening. Based on the cut-offs used for the initial H_1_R virtual screening a set of only 318 compounds was selected for the combined scoring approach (Table S2). The consistency filter was not applied due to the low number of remaining compounds. A less strict novelty filter than on the H_1_R screening was applied (ECFP-4 Tanimoto score of ≤0.5 compared to any known ß_2_R ligand) as the many known ß_2_R ligands show limited chemical diversity^13^,^25^. The compounds for each of the individual scoring methods were selected based on a similar procedure as for the H_1_R, after which the top 750 scoring compounds for each scoring approach were selected. The final compounds were selected after MACCS and visual clustering of the compounds based on scaffold similarity for each of the scoring methods. During the final selection process priority was given to compounds with a lower ECFP-4 score. It should be noted that despite our efforts to perform this selection process systematically, the difference in size between the individual scoring selections (750 compounds) versus the combined scoring selection (318 compounds) might have introduced a positive bias for the individual approaches during the final compound selection as there was a larger pool of compounds to select from.

In total 63 compounds (Fig. 1) were purchased and experimentally tested for their ability to increase GPCR signalling measured by a cAMP response element (CRE) controlled luciferase reporter gene assay in β_2_R-transfected HEK293T cells. Table 2 gives an overview of the results and Fig. 2b illustrates the robustness of the assays performed.

#### Dissecting the contribution for each of the scoring methods

It has been frequently claimed that the combination of two virtual screening approaches resulted in increased performance in retrospective evaluations^14^,^15^ and prospective applications^16^,^17^,^18^. However, in these prospective applications the performance of the *individual* virtual screening approaches has, to the best of our knowledge, never been experimentally validated to allow true comparison of combined and individual virtual screening methods. Here we have validated both our combined PLANTS-IFP scoring approach as well as the individual IFP and PLANTS scoring functions on two different test cases in order to assess if the combination actually performs better than the individual approaches and is not the result of overfitting by retrospective optimization. The systematic evaluation of virtual screening strategies has enabled us to: i) assess the impact of the consensus and single scoring methods on chemical diversity and novelty of the hits, ii) to estimate the target-dependent performance of the approaches, and iii) to dissect the contributions of the individual scoring methods to the consensus method.

The experimental validation of the compounds (Fig. 2 and Supplementary Fig. S2) showed that, although the combined-scoring approach was the most successful, the individual approaches resulted in high hit-rates as well. For the H_1_R VS hit rates of 73%, 61%, and 45% were obtained for the combined, IFP, and PLANTS approach respectively (Tables 1 and 2, Fig. 3a,c). For the β_2_R VS the hit rates were 53%, 44%, and 39% for the combined, IFP, and PLANTS approach respectively (Table 2, Fig. 3b,c). In short, both virtual screens show the same trends: the combined IFP and PLANTS scoring approach is most successful, followed initially by IFP-score ranking and finally by PLANTS-score ranking.

**Figure 4:**
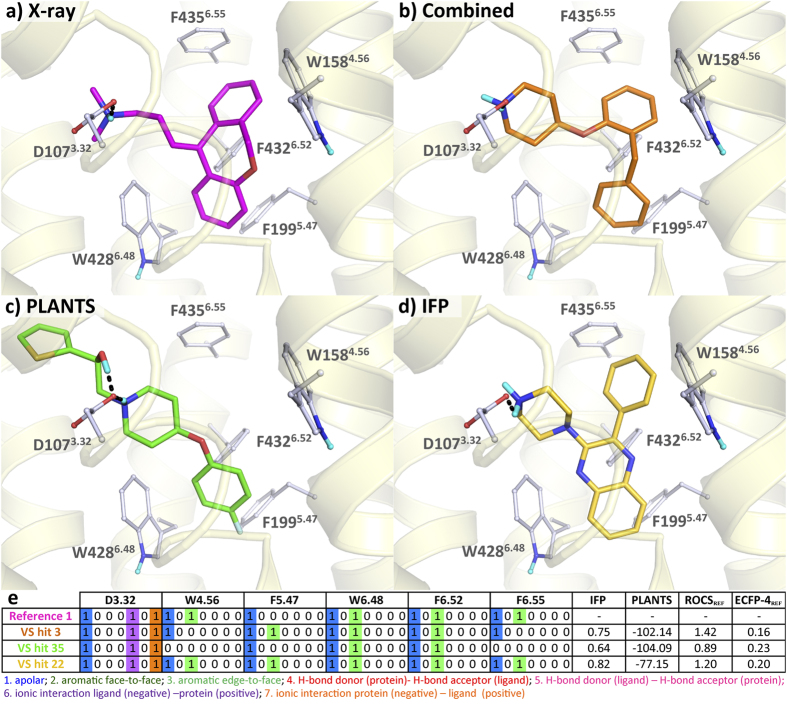
Proposed binding modes of a H_1_R hit from each of the scoring approaches compared to the X-ray structure. Binding modes in H_1_R of (**a**) the co-crystallized **1** (magenta carbon atoms), (**b**) combined PLANTS-IFP-scoring hit **3** (orange carbon atoms), (**c**) PLANTS-scoring hit **35** (green carbon atoms), and (**d**) an IFP-scoring hit **22** (gold carbon atoms). (**e**) The interaction fingerprints of the compounds with each of the depicted residues.

The compound sets for each of the individual scores overlap partially with the compound sets of the combined scoring approach as is illustrated by the Venn diagrams in Fig. 3d. By breaking the compounds sets (and thus hit-rates) down into unique subsets, the contribution of each approach individually can be derived more accurately (Fig. 3c,d) as the differences in hit-rates between the individual approaches are subtle (specifically for the β_2_R screening). Interestingly, the majority of the β_2_R hits that were identified in the individual IFP and PLANTS selection were also identified by the combined approach as well (11 of 16 and 8 of 11 hits respectively). Although similar results were observed in the H_1_R screening, they were less pronounced (7 out of the 20 hits and 4 of 15 hits respectively). This can mainly be ascribed to the fact that the distribution of the IFP and PLANTS scores for the docked compounds differ greatly between the H_1_R and β_2_R screening, as can be derived from the IFP versus PLANTS scoring scatterplots in Fig. 1. Overall the PLANTS scores are higher for the H_1_R than for the β_2_R and the IFP scores are more widely spread for the H_1_R (Fig. 1, Table S2). For the IFP scores this can be explained by the difference in size of the reference ligand of both targets. Compound **1** (Fig. 4a) is a fragment-like ligand with only 21 heavy atoms, thereby matching the size of the compounds in the screening library. Compound **47** on the other hand contains 27 heavy atoms thereby making more contacts and interactions than the fragments in the screening library are able to make based on their size. This discrepancy in size logically results in a reduced IFP-score for the compounds in the fragment-library. In the future this effect could be addressed by e.g. taking a different reference ligand using a fragment of the original reference ligand or by using a different similarity coefficient. The PLANTS scoring differences represent the differences in the shape and composition of the binding site^36^, thereby clearly demonstrating that each protein target benefits from a customized SBVS approach even if two targets are closely related like these two aminergic GPCRs. Thorough retrospective validation and optimization of a virtual screening method is therefore essential to maximize the potency of a method during a prospective application. It must, however, also be recognized that customization of a VS approach often introduces a subjective element (as do the visual clustering and visual inspection steps).

The percentages of hits versus inactives for sets matching the combined scoring criteria are consistently higher (Fig. 3c). Moreover, of the tested H_1_R compounds that are unique to the combined scoring function (section 1 in Fig. 3d) 84.6% were found to be active. On the other hand, for the β_2_R screening most of the hits (16 of the 26 identified hits) were in the individual IFP selection (sections 3 and 4 in Fig. 3d) of which 69% also intersected with the combined scoring approach. This emphasizes once more that the presence of active compounds compared to inactive compounds is higher in the compound selection based on the combined scoring, as was hypothesized based on the retrospective H_1_R virtual screening study^21^. Other successful, non-GPCR, examples of prospective IFP-driven virtual screening studies such as for the Trypanosoma Brucei phosphodiesterase B1^37^ and ligand-gated ion channel GABAA^38^ also demonstrate the added value of using IFP for the hit identification. Moreover, another advantage from a medicinal chemistry point of view is that the hits obtained through the (combined) use of IFP have reliable predicted binding modes as they are similar to previously observed and experimentally validated binding modes. These binding modes can serve as starting points for further ligand optimization efforts and can even be effectively combined with interaction-annotated chemogenomics databases (such as KLIFS^39^ for kinases and PDEStrIAn^40^ for phosphodiesterases) to drive optimization programs^37^ in order to target specific subpockets or to obtain interaction patterns associated with a specific functional effect^22^.

A comparative analysis of 29 GPCR structure-based virtual screening studies^6^,^17^,^41^,^42^,^43^,^44^,^45^,^46^,^47^,^48^ shows that the hit rate of the combined PLANTS-IFP H_1_R virtual screening study (73%) is the highest reported and the percentage of submicromolar-affinity hits (27%) is amongst the highest reported (the average submicromolar hit rate was 7%), together with the SBVS studies on the 5-HT_1_B serotonin receptor^41^ (36%), the α_1A_-adrenoceptor^48^ (30%), and the D_3_ dopamine receptor^17^ (40%).

#### Diverse scaffolds with conserved binding modes

Both virtual screening studies have resulted in the identification of novel and (relatively) potent fragment-like small molecules. The ligands identified with the combined or the IFP approach have a high IFP score indicating that they are expected to have similar interactions within the binding pocket, which translates to comparable binding modes obtained with diverse scaffolds. For H_1_R for example, hits **3**, **22**, and **1** (as shown in Fig. 4) occupy almost the same space in the binding site and make comparable interactions with the pocket residues despite their different molecular structure. However, many of the H_1_R hits that are unique for the PLANTS scoring method, e.g. **35** (Fig. 4c), **37**, **40**, and **45**, are linear ligands (shape-wise) that therefore also address part of the minor pocket (between TM 2, 3, and 7) according to their predicted binding mode, unlike **1**. This might be the result of the docking scoring function as molecules with more protein-ligand contacts (artificially) obtain higher interaction scores^19^, whereas molecules selected with IFP are ligands that adopt a similar binding mode (and thus shape) as the reference ligands. It should also be noted that the use of IFP can compensate for the fact that the PLANTS docking was performed with a rigid receptor (except for the hydroxyl groups, which could freely rotate). The PLANTS score does therefore not take binding site flexibility into account that could potentially compensate for less optimal binding poses, while the use of IFP is able to select compounds that do meet the IFP requirements while it prevents potential over-fitting of the pocket to accommodate the ligand docking pose. Since the reference molecule for the β_2_R screening, **47**, is larger than the reference molecule for the H_1_R screening **1** and already addresses both the major (between TM 3, 5, 6, and 7) and the minor pocket, this effect is not observed for the β_2_R hits.

Interestingly, an analogue of the most potent hit from our previous study^21^ (**3**, pK_i_ = 8.20, Fig. 4b), fragment **42**, was selected using PLANTS scoring. In this analogue the piperidine is substituted on the 3-position instead of the 4-position and the benzyl has shifted from the 2 to the 4 position of the phenoxy moiety, resulting in a 400-fold lower affinity. Another hit obtained using PLANTS scoring is the bulky compound **36**, which fully occupies the aromatic region between TM5 and TM6^35^ with its 9,10-dihydro-9,10-ethanoanthracene moiety. The most potent H_1_R hits that were identified are **3** (pK_i_ = 8.20, Fig. 4b), **22** (pK_i_ = 7.05, Fig. 4d) and **35** (pK_i_ = 6.97, Fig. 4c) for the Combined, IFP and PLANTS scoring approach respectively.

**Figure 5:**
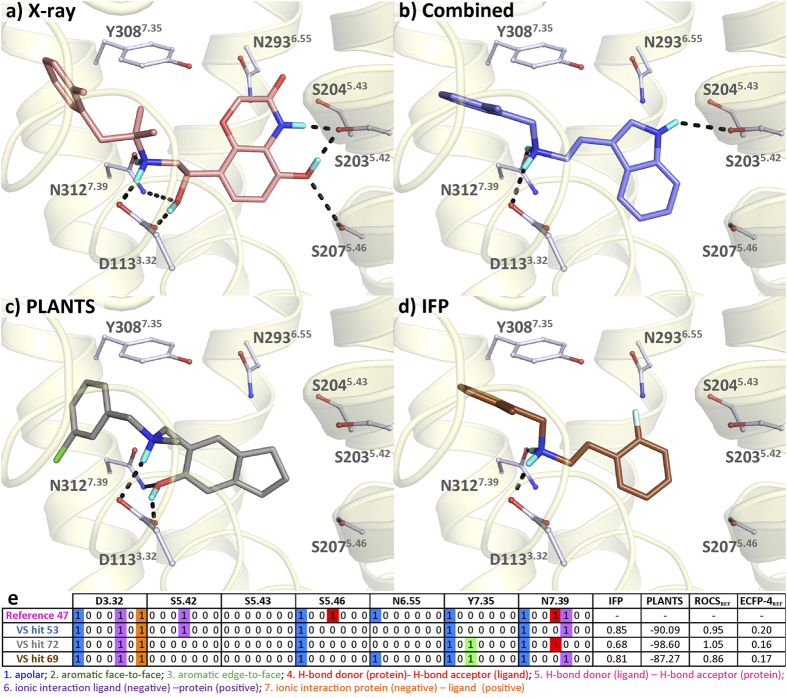
Proposed binding modes of representative β_2_R hits from each of the scoring approaches compared to the X-ray structure. Binding modes in β_2_R of (**a**) the co-crystallized **47** (salmon carbon atoms), (**b**) combined PLANTS-IFP-scoring hit **53** (slate carbon atoms), (**c**) PLANTS-scoring hit **72** (grey carbon atoms), and (**d**) an IFP-scoring hit **69 (**brown carbon atoms). (**e**) The interaction fingerprints of the compounds with each of the depicted residues.

For β_2_R the well-known ethanolamine scaffold is prevalent within the hits (e.g. **50** and **66**), however, more than 50% of all identified agonists do not have this scaffold (e.g. **53** and **69**, Fig. 5b,d). Interestingly, a few ligands contain groups that are able to form a similar H-bond interaction network with N312^7.39^ as the conserved ethanolamine alcohol moiety (e.g. **72**, Fig. 5c). It should be noted that Christopher *et al*.^49^ also reported the identification (and subsequent crystallization with β_2_R) of fragment-like β_2_R ligands without an ethanolamine moiety using biophysicial fragment screening. It was suggested by the authors that these fragment-like ligands were expected to be antagonists^49^. Hit **53** forms an H-bond with S203^5.42^ via its indole moiety (Fig. 5b). Interestingly, although this indole moiety is also observed in known β_2_R ligands like pindolol, the indole of **53** is substituted at the 3-position instead of the 4-position (e.g. hit **54**). More surprising is that most of the identified agonists do not contain hydrogen-bond donors or acceptors for interacting with the serines in TM5, which were previously deemed essential for activation^8^,^25^,^32^,^34^,^50^. However, some of the identified agonists do contain halogen-substituents (e.g. **50** and **52**) that could allow for halogen bonding. Strikingly, compounds like **66** are able to increase cAMP formation but do not contain any H-bonding partners for the serines in TM5 but do have a short linker to the aromatic head-group that was previously proposed to play a role in inducing the active-state via aromatic stacking with aromatic residues in TM6^51^. **61** has a 2-(methylamino)-1-phenylethan-1-ol scaffold similar to **66** and **58**. Despite the different substituents on this scaffold for **66** (2,4,6-trimethylbenzyl), **61** (methyladamantane), **58** (benzonitrile) all have comparable potencies (pEC_50_ around 6). However, compound **66** has a significantly higher efficacy (E_max_ 85% ± 6) compared to the other compounds (E_max_ ~60%). Based on the binding modes of these ligands (data not shown) this indicates that not only substitutions near TM5 and TM6, but also between TM3 and TM7 can result in a gain in efficacy. Interestingly, **58** is similar to **49** without the ethanolamine moiety. Their binding modes (not shown), however, are very different as the benzonitrile of **49** interacts with S203^5.42^ whereas it interacts with W313^7.40^ for **58**.

The agonist with the highest potency of all identified β_2_R agonists (pEC_50_ = 7.42), **48**, was identified using the combined approach. However, due to the relatively high similarity of **48** to the well-known β_2_R agonist salbutamol (ECFP-4 similarity = 0.48) this is not surprising. The more novel agonists **50** and **71** have the highest potencies (pEC_50_ = 6.73 and pEC_50_ = 6.81) of the hits from the IFP and PLANTS approach, respectively.

It should be noted that the potency of isoprenaline as observed in these assays is relatively low (pEC_50_ = 6.51), which might be due to oxidation of the catechol moiety. We tried to address this using ascorbic acid (as was previously proposed^52^) but this unfortunately interfered with the reporter gene assay. It is noteworthy that the potency of isoprenaline varies throughout scientific publications and seem to be highly assay dependent, as previously reported potencies (pEC_50_) for isoprenaline range from 6.62^53^ to 10.1^13^.

The combined scoring approach did select the compound with the highest affinity for H_1_R and potency for β_2_R. Despite this, there does not seem to be a clear correlation between affinity (pK_i_), potency (pEC_50_), and efficacy (E_max_) when analysing the hits from the three selection approaches (Fig. 3a,b). Based on the ECFP-4 similarity compound **28** is the most novel hit for H_1_R. Although the structure of compound **28** is different from most known H_1_R ligands, the typical H_1_R pharmacophore elements can still be recognized. Compound **43**, the second most novel H_1_R hit, on the other hand, has a very different scaffold with its furan, tetrahydrofuran and chlorobenzene moieties. From the identified β_2_R hits compound **56** is the most novel hit according to its ECFP-4 similarity to known β_2_R ligands. Interestingly, **56** has a similar scaffold as the novel H_1_R ligand **43** but the tetrahydrofuran group has been replaced with a thiophene and the chlorine atom was removed.

**Figure 6:**
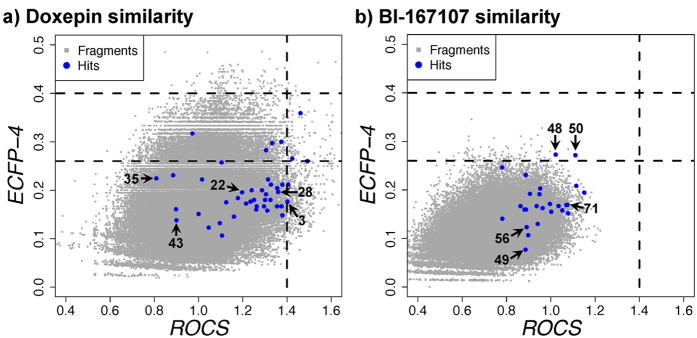
2D/3D ligand-based virtual screening on the fragment library using (**a**) the H_1_R reference ligand **1** and (**b**) the β_2_R reference ligand **47**. The experimentally validated hits (blue) as well as all screened fragments (grey) are scattered based on their ECFP-4^24^ and ROCS similarity to the doxepin reference ligand. The horizontal dotted line indicates the cut-off^21^ of 1.4 for the ROCS ComboScore. The vertical dotted lines indicate the cut-offs^21^ of 0.26 and 0.40 for ECFP-4.

When comparing the entire screening library to the co-crystallized reference ligand using 2D topological (ECFP-4) and 3D shape-based (ROCS) similarity searches we observe two very different distributions for the targets (Fig. 6). This can be ascribed to the differences in size, shape, and pharmacophore features of the two reference ligands, **1** and **47**. As **1** itself also matches the fragment-like criteria of the screening library the overall similarity is much higher (Fig. 6a). The higher heavy atom count and thus volume of **47** results in a generally lower ECFP-4 and ROCS scores for the compounds from the fragments library (Fig. 6b). For the H_1_R hits only 3 of the identified ligands were present in the top 500 as selected by ROCS and 0 as selected by ECFP-4 (Fig. 6a). For β_2_R 7 and 4 of the ligands were present in the top 750 based on ROCS and ECFP-4 scoring, respectively, albeit with low similarity scores (Fig. 6b).

We furthermore assessed the novelty of the identified ligands by performing SEA^54^ predictions (functional and binding) and the ChEMBL target prediction models in myChEMBL^55^ (using both the 10 μM and 1 μM models). The ChEMBL models predicted 1 of the 26 β_2_R SBVS hits as a human β_2_R ligand, another 2 hits as human β_1_R ligands and 4 of the 43 H_1_R SBVS hits as human H_1_R ligands. SEA (binding and functional combined) predicted 1 of the 26 β_2_R SBVS hits as a human β_2_R ligand, 2 as human β_1_R ligands and another 2 as animal β_2_R ligands, and none of the 43 H_1_R SBVS hits as H_1_R ligand.

#### Predicting both ligand binding affinity and functional activity

The β_2_R screening tries to combine the identification of new compounds with affinity for β_2_R as well as predicting the functional activity by only searching for β_2_R agonists. Only limited studies with a similar goal have been performed on GPCRs of which most were retrospective analyses^8^,^25^,^50^. However, as described in the introduction, more recently a prospective virtual screening for β_2_ as well as D_2_ receptor agonists was published using the same active-state β_2_R structure (PDB 3P0G) as used in this study. Weiss *et al*. performed a systematic prospective study to investigate the effect of receptor conformation on virtual screening^13^. In this study, a library of 2.7 million lead-like and 0.4 million fragment-like molecules from the ZINC database was screened against the active-state **47**-bound β_2_R crystal structure (PDB 3P0G) and carazolol-bound crystal structure (PDB 2RH1)^13^. During the docking the dipole moment of S203^5.42^, S204^5.43^, or S207^5.46^ was increased to augment docking scores for poses in polar contact with these residues. Compounds ranking within the top 0.2% of the active-state structure and ranking at least 5000 positions higher for the active-state compared to the carazolol-bound structure were selected for further processing. Compounds that had at least a positive charge, an ionic interaction with D113^3.32^ and at least one H-bond with any of the three aforementioned serines were visually inspected. In total 5 fragment-like and 17 lead-like molecules were experimentally validated resulting in the identification of 1 fragment-like and 5 lead-like β_2_R agonists, of which 1 known β_2_R agonist and 3 compounds with an ECFP-4 similarity higher than 0.4. Moreover, based on a homology model of an active-state D_2_ receptor a similar virtual screening was performed with the aim to identify new D_2_ agonists. 15 compounds were selected for experimental validation of which 3 were found to be hits with marginal potencies (of which 2 with an ECFP-4 similarity higher than 0.4): 2 agonists and 1 antagonist.

Although most structure-based virtual screenings tend to identify ligands with the same functional effect as the co-crystallized ligand or the ligand(s) used to refine a homology model^21^,^43^,^44^,^45^,^46^, there have been multiple prospective virtual screenings based on homology models of GPCRs that have resulted in the identification of ligands with a different functional effect^13^,^17^,^42^,^47^.

Despite the difficulties that come with these types of predictions, as illustrated by the examples above, we were able to selectively identify 26 β_2_R agonists. It should be noted that the β_2_R was also the ideal receptor for such an analysis, as much information is available for this receptor including (and most importantly) multiple crystal structures in different activation states and in complex with both agonists and antagonists/inverse agonists^5^,^9^,^26^,^27^,^28^,^29^,^30^,^31^,^32^,^33^,^34^. Generally, aminergic GPCRs have a deep and well-defined binding site without a large solvent-exposed area which make them suitable for docking simulations as demonstrated by other successful prospective structure-based virtual screening studies against aminergic GPCR crystal structures^6^,^17^,^41^,^44^,^45^,^46^,^48^. Other GPCRs such as, for example, the chemokine receptors CXCR4^56^ and CCR5^57^, have a larger and more open pocket, which provide challenges with respect to conformational sampling in molecular docking simulations and subsequent scoring of docking poses, and structure-based virtual screening against chemokine receptors crystal structures have so far resulted in lower hit rates of larger hits with lower affinity^6^,^43^,^58^. Moreover, for many GPCRs structure-function relationships are not as well defined as for beta-adrenergic receptors^59^, thereby preventing the training of predictive models such as the one presented in the current study. On the other hand, the growing amount of GPCR crystal structures in complex with different ligands will provide structural information to guide and optimize ligand function-specific structure-based virtual screening studies against more and more GPCR targets. At this point in time, function-selective structure-based screenings such as described here are already in reach for the A_2A_^60^, M_2_^61^, P2Y_12_^62^, and μ-opioid^63^ receptor for which both small molecule agonist and antagonist/inverse agonist bound crystal structures are available. As the insights for the different signalling pathways that ligands can block or induce is growing^64^ (together with the aforementioned availability of GPCR crystal structures) this could also open up new opportunities^65^ to finding ligands portraying specific biased signalling profiles^4^,^66^.

## Conclusions

Conventional docking approaches are hampered by challenges in the prediction of the right binding mode and the correct ranking of those binding modes. To overcome these hurdles we have devised a novel docking scoring approach that combines a conventional energy-based scoring function with an interaction-profile-based rescoring approach. This approach was successfully applied by prospectively screening a fragment-like compound library on two GPCRs for which crystal structures are available. For both the H_1_ and β_2_ receptor high hit-rates were obtained. Moreover, besides the consensus approach also the individual approaches were experimentally validated in order to evaluate if the combination indeed resulted in an increased performance (hit-rates) in a prospective manner. Although the individual scoring approaches were effective as well, the combined approach did result in increased hit-rates and the retrieval of ligands with up to nanomolar affinities and potencies. It should also be noted that despite the fact that the combined scoring approach was proven to be effective for multiple protein targets, the scoring distributions for the targets did highlight a highly-target-specific score distribution due to the pocket composition and the impact of the reference ligand. It is therefore recommended to optimize this combined approach for each targeted protein using careful retrospective validation to e.g. optimize the docking scoring function and IFP scoring cut-offs. Accordingly, if multiple crystal structures are available, the selection of the right structure (or combination of structures^13^) will influence the outcome of the virtual screening in terms of retrieval rates but also functional effect of the obtained hits. For the β_2_R we were able to selectively retrieve novel fragment-like ligands with the desired functional effect. Moreover, this approach led to the identification of novel scaffolds for β_2_R agonists. Surprisingly, some of these hit compounds did not contain hydrogen bond acceptors or donors that would be able to interact with serines S203^5.42^, S204^5.43^, or S207^5.46^ in TM5, previously thought to be crucial for β_2_R activation^8^,^25^,^32^,^34^,^50^. The results show that the advances in GPCR crystallography open up new opportunities to selectively discover new GPCR ligands with the desired functional effect. These advances could ultimately lead to the prediction and structure-based optimization of ligands with designed (biased) signalling profiles^4^,^65^,^66^.

## Experimental Procedures

### Residue numbering and nomenclature

The Ballesteros–Weinstein residue numbering scheme^67^ was used throughout this manuscript. For residues in specific receptors, the UniProt residue number is given before the Ballesteros–Weinstein residue number in superscript (e.g. D107^3.32^ in H_1_R).

### Preparation of prospective virtual screening database

The compound libraries of 15 vendors were obtained from the ZINC database in SMILES format totaling ~13 million unique compounds. Openeye’s filter (version 2.1.1) was used to only select fragment-like compounds were resulting in a subset of 757.728 compounds. Tauthor (version 1.4.90) and Blabber (version 1.4.90) from MolDiscovery’s MoKa package were used to compute plausible tautomers and protonation states. Subsequently, another filter was applied to remove all compounds without a positive formal charge to ensure only compounds, which could potentially form an ionic bond with key residue D^3.32^ were selected. These steps resulted in a final prospective virtual screening library comprising 108 790 compounds.

### Automated docking and IFP post-processing

The dockings were performed using PLANTS^19^ and the resulting H_1_R and β-adrenergic docking poses were post-processed and ranked with IFP^20^,^21^. Using PLANTS 25 docking poses for each compound were calculated (speed setting 2) and scored using the ChemPLP scoring function. The docking site of β_2_R and H_1_R was defined by the coordinates of the center of cocrystallized ligand (BI-167107 and doxepin, respectively) and a radius around it based on the maximum distance from this center to the edge of the crystallized ligand +5 Å. All other options of PLANTS were left at their default setting. PLANTS employs an ant-colony-optimization algorithm for the prediction of binding poses of small molecules in a protein structure and an empirical scoring function, ChemPLP, for the scoring of the resulting binding poses. IFP evaluates a (predicted) binding mode of a compound in a protein structure by annotating the absence or presence of different types of interactions (hydrophobic, aromatic, H-bond, ionic) between each pocket residue and the molecule based on a set of rules^20^. This results in a molecular interaction fingerprint representing all interactions between the molecule and the protein in bit-string, allowing for the easy comparison and scoring (using the Tanimoto coefficient) of the similarity of multiple IFPs. The H_1_R and β-adrenoceptor pockets were defined by 33 pocket residues based on the consensus pocket definition by de Graaf *et al*.^25^: L/M^1.35^, L/M^1.39^, I^1.42^, T/I^1.46^, V^2.57^, M/V^2.58^, N/G^2.61^, L/I^2.65^, W^3.28^, L/T^3.29^, D^3.32^, Y/V^3.33^, S/V^3.36^, T^3.37^, I^3.40^, W/T^4.56^, I/P^4.60^, F/Y^5.38^, K/A^5.39^, T/S^5.42^, A/S^5.43^, N/S^5.46^, F^5.47^, F^6.44^, W^6.48^, Y/F^6.51^, F^6.52^, F/N^6.55^, H/Y^7.35^, I/N^7.39^, W^7.40^, Y^7.43^, N^7.45 ^21. In the β_1_R/β_2_R retrospective validation the binding mode of the co-crystallized compound for each respective crystal structure was used for the calculation of the reference IFP. These reference IFPs were subsequently used to score the docking poses.

### Retrospective virtual screening databases and analysis

The H_1_R retrospective validation was performed by docking a compound library comprising 543 known H_1_R ligands from ChEMBL, 59 CNS active drugs acting as inverse agonists on H_1_R, and 7 088 physicochemically similar decoy molecules into the H_1_R crystal structure. Subsequently all docking poses were postprocessed using IFP and only docking poses in which the compounds made an ionic interaction with D107^3.32^ were analyzed. For each compound the best PLANTS and the best IFP score were selected and used for defining the score cut-offs for prospective application. With a PLANTS score cut-off of ≤−90 and an IFP score cut-off ≥0.75 high enrichment factors for the ligands over the decoys were obtained (EF_1%_ of 39.3 and 57.6 for the ChEMBL and CNS H_1_R ligands respectively)^21^. The test set by de Graaf *et al*.^25^ was used for the retrospective virtual screening study on all 6 β_1_R and 7 β_2_R crystal structures available at that time (PDB accessed May 2011). This test set exists of 13 agonists, 13 antagonist/inverse agonists, and 980 physicochemically-similar decoys and was extended with 7 agonists and 8 antagonists/inverse agonists from Baker *et al*.^68^. From each β-adrenergic crystal structure one chain was selected and used for the retrospective validation. The test set was docked into all selected chains and scored using PLANTS and IFP. Subsequently, the enrichment factors at a 1% false positive rate for the f/pAGO and ANT/iAGO over decoys were determined for each of the crystal structures (Supplementary Fig. S1).

### Prospective virtual screening

The PLANTS and IFP scoring cut-offs (as described in the previous paragraph) were used for prospective virtual screening of a library of 108 790 fragment-like basic molecules^21^ against both H_1_R and β_2_R targets. For the combined scoring approach the filtering was performed by applying the D^3.32^ interaction filter, the PLANTS and IFP cut offs, the consistency cut off in case of H_1_R (only compounds with an IFP score of ≥0.7 according to the best PLANTS pose as well as a PLANTS score of ≤−75 according to the best IFP pose were kept), and a novelty filter (ECFP-4 ≤ 0.4 for H_1_R and ECFP-4 ≤ 0.5 for β_2_R). H_1_R and β_2_R hit selection consisted of a visual clustering of the remaining compounds, after which from each cluster the fragment with the highest IFP and/or PLANTS score was selected and fragments with docking poses in which polar atoms were buried in hydrophobic parts of the binding site were discarded^21^. For the individual PLANTS and IFP scoring approaches the top 500 (H_1_R) and top 750 (β_2_R) scoring compounds were selected, and compounds matching the combined approach were removed. Hits that were similar to any known ligand of the respective H_1_R (ECFP-4 Tanimoto score of 0.4) or β_2_R (ECFP-4 Tanimoto score of ≥0.5) receptors were removed, compounds were visually clustered based on scaffold similarity, and compounds with buried polar groups that were placed in hydrophobic parts of the receptor binding site were discarded by visual inspection.

### ROCS 3D similarity search

The conformer database was generated using standard settings OMEGA (version 2.3.2; OpenEye Scientific Software: Santa Fe, NM.) and searched with ROCS (version 2.3.1; OpenEye Scientific Software: Santa Fe, NM.) using standard settings as well. The conformation of **1** found in the H_1_R X-ray structure (PDB-code 3RZE^23^) and the conformation of **47** in the β_2_R X-ray structure (PDB-code 3P0G) were used as query molecules for independent ROCS runs. The compounds from the screening library were ranked by decreasing Comboscore (combination of shape Tanimoto and the normalized colour score in this optimized overlay).

### ECFP-4 2D similarity search

Two-dimensional similarity searches were carried out using ECFP-4 (extended connectivity fingerprints^24^) descriptors available in Pipeline Pilot (version 6.1.5; Accelrys Software Inc.: 5005 Waterridge Vista Dr, San Diego, CA 92121, United States.) and compared using the Tanimoto coefficient.

### Compounds selected by virtual screening

The compounds selected by virtual screening were purchased from available screening collections of six vendors: Asinex (www.asinex.com), Chembridge (www.Hit2Lead.com), Enamine (www.enamine.com), IBScreen (www.ibscreen.com), Matrix Scientific (www.matrixscientific.com), Vitas-M (www.vitasmlab.com). Suppliers and supplier identifiers for each of the virtual screening hits are given in Table S3. The purity of all compounds was verified by liquid chromatography-mass spectrometry (LC-MS), all experimentally validated hits had a purity of 90% or higher (see Table S4), except compounds **40**, **26**, and **44**, which in our hands had a purity of 75%, 76%, and 88% respectively (reported to be at least 90% pure according to the suppliers).

### Materials

Human H_1_R cDNA was kindly provided by Dr. H Fukui (Japan). The cDNA clone for Human β_2_R in pcDNA3.1+ was obtained from Missouri S&T cDNA Resource Center (www.cdna.org). Cell culture media were purchased from PAA (Pasching, Austria). Isoproterenol was bought from Sigma-Aldrich (St. Louis, MO, USA). Compounds used in the assays were obtained from different suppliers (see Supplementary Table S4). The cDNA clone for Human β_2_R in pcDNA3.1+ was obtained from Missouri S&T cDNA Resource Center (www.cdna.org).

### Cell culture and transfection

The cell culture and transfection for H_1_R was performed as previously described^21^. HEK293T cells were cultured in Dulbecco’s modified Eagle medium (DMEM) supplemented with 10% fetal bovine serum, 50 IU/ml penicillin and 50 μg/ml streptomycin at 37 °C and 5% CO_2_. Approximately 2 × 10^6^ million cells were seeded per 10-cm dish 1 day prior to transfection. Approximately 4 × 10^6^ cells were transiently transfected with 5 μg of cDNA using 25 the polyethylenimine (PEI) method. Briefly, 10 ng β_2_R cDNA, 2,5 μg CRE-luc plasmid, and 2.490 μg empty pcDNA3.1 were mixed with 20 μg of 25 kDa linear PEI (Polysciences, Warrington, PA, U.S.) in 500 μL of 150 mM NaCl. The transfection mix was incubated at 22 °C for 30 min. Meanwhile, medium in the 10 cm dish was replaced with fresh culture medium and transfection mix was subsequently added drop-wise to the cells. The next day, cells were collected and transferred to white-bottomed 96-well plates (50,000 cells/well).

### Radioligand displacement assay

The radioligand displacement assay for H_1_R was performed as previously described^21^.

### CRE (cyclic AMP response element) luciferase reporter gene assay

Two days after transfection, the medium was removed and the cells were stimulated for 6 h with ligands in serum-free DMEM supplemented with 1 mM thiourea to slow the oxidation of compounds, at 37 °C, 5% CO_2_. After 6 h, the medium was aspirated and 25 μl of luciferase assay reagent (LAR, 0.83 mM ATP, 0.83 mM D-luciferine, 18.7 mM MgCl_2_, 0.78 μM Na_2_HPO_4_, 38.9 mM Tris-H_3_PO_4_ (pH 7.8), 0.39% glycerol, 0.03% Triton X-100 and 2.6 μM dithiotreitol) was added to each well. Luminescence (1 s per well) was measured in a Victor^3^ 1420 multi-label reader (Perkin Elmer Life and Analytical Sciences) after 30 min of incubation at 37 °C, 5% CO_2_. Non-linear curve-fitting and statistical analysis were performed using GraphPad Prism 6. Results are shown from pooled data (mean ± SEM) from at least three independent experiments performed in triplicate.

## Additional Information

**How to cite this article**: Kooistra, A. J. *et al*. Function-specific virtual screening for GPCR ligands using a combined scoring method. *Sci. Rep.* **6**, 28288; doi: 10.1038/srep28288 (2016).

## Supplementary Material

Supplementary Information

## Figures and Tables

**Table 1:**
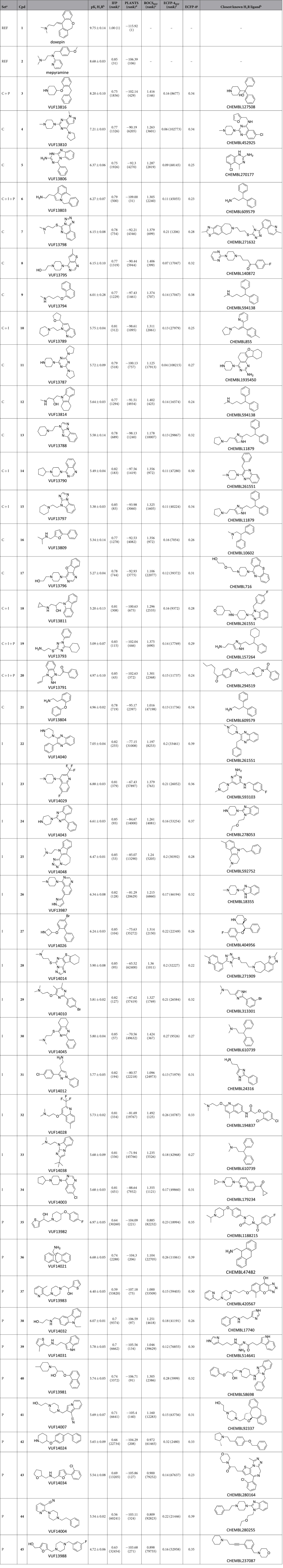
Experimentally validated H_1_R hits based on the IFP and PLANTS approach.

^a^The letters C, I, and P, refer to the distinct compound selections from each the Combined, IFP, and PLANTS scoring approach, respectively. Combinations like C + I indicate that the compound was present in both the selection of the Combined and the IFP scoring approach but not the PLANTS approach. Further details are shown in Fig. 3. ^b^p*K*_i_ values are calculated from at least three independent measurements as the mean ± SEM. Measured by displacement of [^3^H]-mepyramine binding using membranes of HEK293T cells transiently expressing the human H_1_R. ^c^IFP Tanimoto similarity to the pose of doxepin in the H_1_R crystal structure. Optimized IFP score cut-off ≥0.75. IFP ranking is given between brackets. ^d^Score and rank according to PLANTS scoring function^19^. Optimized PLANTS score cut-off ≤−90. PLANTS ranking is given between brackets. ^e^ROCS shape-based 3D similarity to doxepin based on Comboscore^69^. ROCS ranking is given between brackets. ^f^ECFP-4 2D Tanimoto similarity to doxepin. A similarity higher than 0.40 is considered as significative^21^. ECFP-4 ranking is given between brackets. ^g^ECFP-4 circular fingerprint Tanimoto similarity to closest known H_1_R active in ChEMBLdb. A similarity higher than 0.40 is considered as significative^21^. ^h^The closest known H_1_R active in the ChEMBLdb as determined by the ECFP-4 similarity.

**Table 2:**
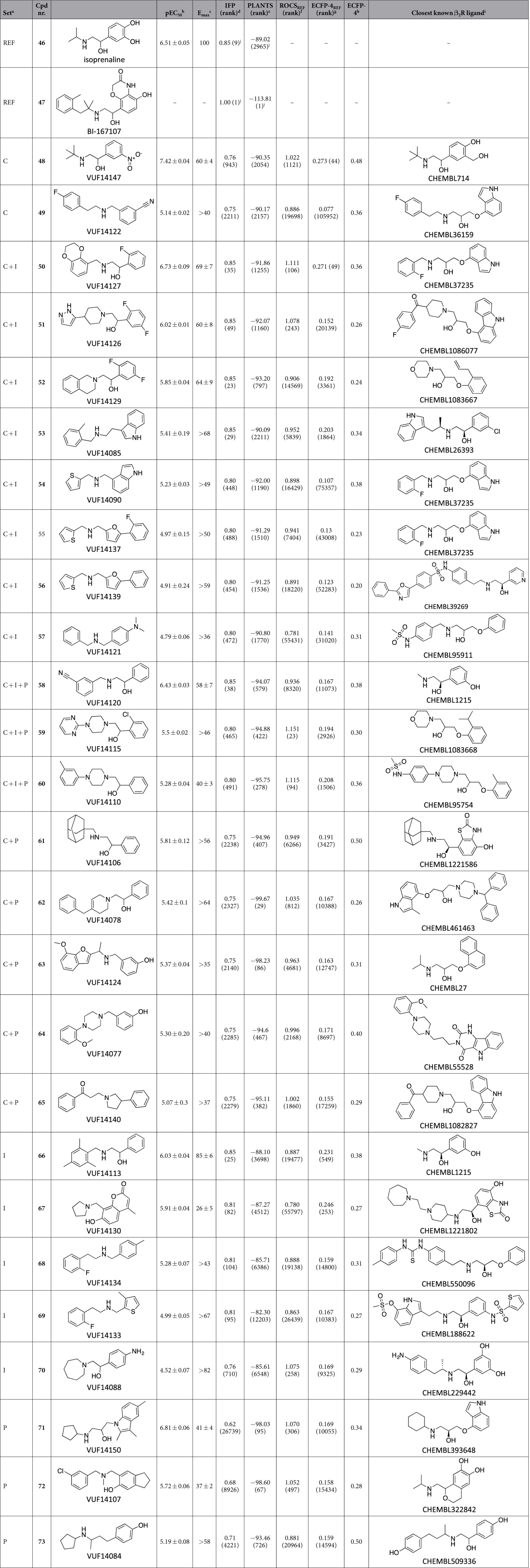
Experimentally validated β_2_R hits based on the IFP, PLANTS and combined approach.

^a^The letters C, I, and P, refer to the distinct compound selections from each the Combined, IFP, and PLANTS scoring approach, respectively. Combinations like C + I indicate that the compound was present in both the selection of the Combined and the IFP scoring approach but not the PLANTS approach. Further details are shown in Fig. 3. REF indicates reference compounds. ^b^pEC_50_ values are calculated from at least three independent measurements as the mean ± SEM. ^c^Maximum CRE-luciferase response values normalized to isoproterenol (100%) and basal activity (0%) using HEK293T cells transiently expressing the human β_2_R. For values preceded by “>” the maximum response plateau was not observed, therefore the response at 100 μM was reported (except for compounds **50**, **53**, **59**, **65**, and **71**, for which the response at 10μM is reported). ^d^IFP Tanimoto similarity to the pose of BI-167107 in the β_2_R crystal structure. Optimized IFP score cut-off ≥0.75. IFP ranking is given between brackets. ^e^Score and rank according to PLANTS scoring function^19^. Optimized PLANTS score cut-off ≤−90. PLANTS ranking is given between brackets. ^f^ROCS shape-based 3D similarity to BI-167107 based on Comboscore^69^. ROCS ranking is given between brackets. ^g^ECFP-4 2D Tanimoto similarity to BI-167107. A similarity higher than 0.40 is considered as significative^21^. ECFP-4 ranking is given between brackets. ^h^ECFP-4 circular fingerprint Tanimoto similarity to closest known β_2_R active in ChEMBLdb. A similarity higher than 0.40 is considered as significative^21^. ^i^The closest known β_2_R active in the ChEMBLdb as determined by the ECFP-4 similarity. ^j^The rankings indicated for reference BI-167107 and isoprenaline were determined as if they were included in the screening library.
