# Supplementary material for: Function-specific virtual screening for GPCR ligands using a combined scoring method
Source: Sci Rep. 2016 Jun 24;6:28288. doi: 10.1038/srep28288 (PMC4919634; doi:10.1038/srep28288)
Supplement: Supplementary Information [file srep28288-s1.pdf]

Supporting information

Function-specific virtual screening for GPCR ligands using a combined scoring method

Albert J. Kooistra, Henry F. Vischer, Daniel McNaught-Flores, Rob Leurs, Iwan J.P. de Esch, Chris de Graaf

Amsterdam Institute for Molecules, Medicines and Systems (AIMMS), Division of Medicinal Chemistry, Faculty of Sciences, Vrije Universiteit Amsterdam, De Boelelaan 1108, 1081 HZ Amsterdam, The Netherlands.

Table S1. Overview of all 32 β-adrenoceptor crystal structures and the co-crystallized ligands.

**Ligand**	**PDB (reference)**
7-methylcyanopindolol	5A8E (Sato et al., 2015[1])
Alprenolol	3NYA* (Wacker et al., 2010[2])
Arylpiperazine 19	3ZPQ (Christopher et al., 2013[3])
Arylpiperazine 20	3ZPR (Christopher et al., 2013[3])
BI-167107	3P0G* (Rasmussen et al., 2011[4])<br>3SN6 (Rasmussen et al., 2011[5])<br>4LDE (Ring et al., 2013[6])
Bucindolol	4AMI (Warne et al., 2012[7])
Carazolol	2YCW (Moukhametzianov et al., 2011[8])<br>2RH1* (Cherezov et al., 2007[9])<br>4GBR (Zou et al., 2012[10])<br>2R4R (Rasmussen et al., 2007[11])<br>2R4S (Rasmussen et al., 2007[11])<br>3KJ6 (Bokoch et al., 2010[12])
Carmoterol	2Y02* (Warne et al., 2011[13])
Carvedilol	4AMJ (Warne et al., 2012[7])
Cyanopindolol	2VT4* (Warne et al., 2008[14])<br>2YCX (Moukhametzianov et al., 2011[8])<br>2YCY (Moukhametzianov et al., 2011[8])<br>4BVN (Miller-Gallacher et al., 2014[15])
Disulfide-functionalized adrenaline analog 2	4QKX (Weichert et al., 2014[16])
Dobutamine	2Y00* (Warne et al., 2011[13])<br>2Y01* (Warne et al., 2011[13])
Epinephrine	4LDO (Ring et al., 2013[6])
FAUC50	3PDS* (Rosenbaum et al., 2011[17])
Hydroxybenzylisoproterenol	4LDL (Ring et al., 2013[6])
ICI 118,551	3NY8* (Wacker et al., 2010)
Iodocyanopindolol	2YCZ (Moukhametzianov et al., 2011)
Isoproterenol	2Y03* (Warne et al., 2011[13])
Ligand-free	4GPO (Huang et al., 2013[18])
Salbutamol	2Y04* (Warne et al., 2011[13])
Timolol	3D4S* (Hanson et al., 2008[19])
VS hit (Kolb)	3NY9* (Wacker et al., 2010[2])

* These structures were used in the retrospective training, see Figure S1.

Table S2. Number of compounds from the fragment library matching the scoring cutoff criteria.

	**Combined**	**IFP ≥ 0.75**	**PLANTS ≤ -90**
H_1R	611	2274	6416
β_2R	318	2331	2278

Table S3. Supplier information for each of the validated compounds.

VUF #	Supplier	Supplier code	VUF #	Supplier	Supplier code
**13981**	Asinex	BAS 04937691	**14078**	ChemBridge	5286420
**13982**	Asinex	SYN 19977907	**14084**	ChemBridge	5464199
**13983**	Asinex	SYN 19978343	**14085**	ChemBridge	5522599
**13987**	ChemBridge	39155140	**14088**	ChemBridge	9123904
**13988**	ChemBridge	62453889	**14090**	ChemBridge	9189424
**14003**	Enamine	T6187031	**14106**	Enamine	T5235450
**14004**	Enamine	T6195896	**14107**	Enamine	T5514695
**14007**	Enamine	T6487446	**14110**	Enamine	T6065278
**14010**	Enamine	T6747647	**14113**	Enamine	T6463568
**14012**	Enamine	EN300-57183	**14115**	Enamine	T6547101
**14014**	IBScreen	STOCK5S-03913	**14120**	Enamine	T6705366
**14021**	Labotest	LT03357026	**14121**	Enamine	T6734476
**14024**	Matrix Scientific	23219	**14122**	Enamine	T6741662
**14026**	Matrix Scientific	49413	**14124**	Enamine	T6762894
**14028**	Maybridge	KM07508	**14126**	Enamine	T6802160
**14029**	Maybridge	KM10688	**14127**	Enamine	T6809874
**14031**	Specs	AN-465/42886205	**14129**	Enamine	T6895647
**14032**	Specs	AN-465/43013625	**14130**	Specs	AF-399/37418022
**14034**	Specs	AN-465/43369264	**14133**	Specs	AN-465/42767321
**14038**	Vitas-M	STK037441	**14134**	Specs	AN-465/42767354
**14040**	Vitas-M	STK205963	**14137**	Specs	AN-465/43369113
**14043**	Vitas-M	STK546007	**14139**	Specs	AN-465/43369482
**14045**	Vitas-M	STK590459	**14140**	Specs	AO-365/15162044
**14048**	Vitas-M	STK741162	**14147**	Vitas-M	STK699950
**14077**	ChemBridge	5269659	**14150**	Vitas-M	STK945008

Table S4. Purity data for each of the validated compounds as measured by LC-MS[a].

VUF #	Purity (%)	VUF #	Purity (%)
**13981**	75*	**14078**	-*
**13982**	94	**14084**	-*
**13983**	96	**14085**	>99
**13987**	76*	**14088**	>99
**13988**	-*	**14090**	98
**14003**	96	**14106**	94
**14004**	88*	**14107**	100[b]
**14007**	100	**14110**	>99[b]
**14010**	91	**14113**	>99
**14012**	98	**14115**	-*
**14014**	97	**14120**	-*
**14021**	92	**14121**	97
**14024**	-*	**14122**	99
**14026**	-*	**14124**	-*
**14028**	100	**14126**	-*
**14029**	>99	**14127**	-*
**14031**	100	**14129**	>99
**14032**	100	**14130**	>99
**14034**	>99	**14133**	>99
**14038**	100	**14134**	>99
**14040**	100	**14137**	>99
**14043**	100	**14139**	>99
**14045**	98	**14140**	-*
**14048**	100	**14147**	96
**14077**	-*	**14150**	>99

[a] Analytical HPLC-MS analyses were conducted using a Shimadzu LC-20AD liquid chromatograph pump system with a Shimadzu SPD-M20A diode array detector. MS detection was performed with a Shimadzu LCMS-2010 EV liquid chromatograph mass spectrometer. The analyses were performed using the following conditions; Xbridge (C18) 5 μm column (50 mm × 4.6 mm) with solvent A (acetonitrile with 0.1% formic acid) and B (water with 0.1% formic acid), flow rate of 1.0 mL/min, start 5% A, linear gradient to 90% A in 4.5 min, then 1.5 min at 90% A, then a linear gradient to 5% A in 0.5 min, then 1.5 min at 5% A, total run time of 8.0 min. Compound purities were calculated as the percentage peak area of the analyzed compound by UV detection at 230 nm

[b] NMR analysis indicated a 10-15% degradation of these compounds

- Not enough material left for LC-MS analysis.

[*] These compounds were reported to be at least 90% pure according to the suppliers.

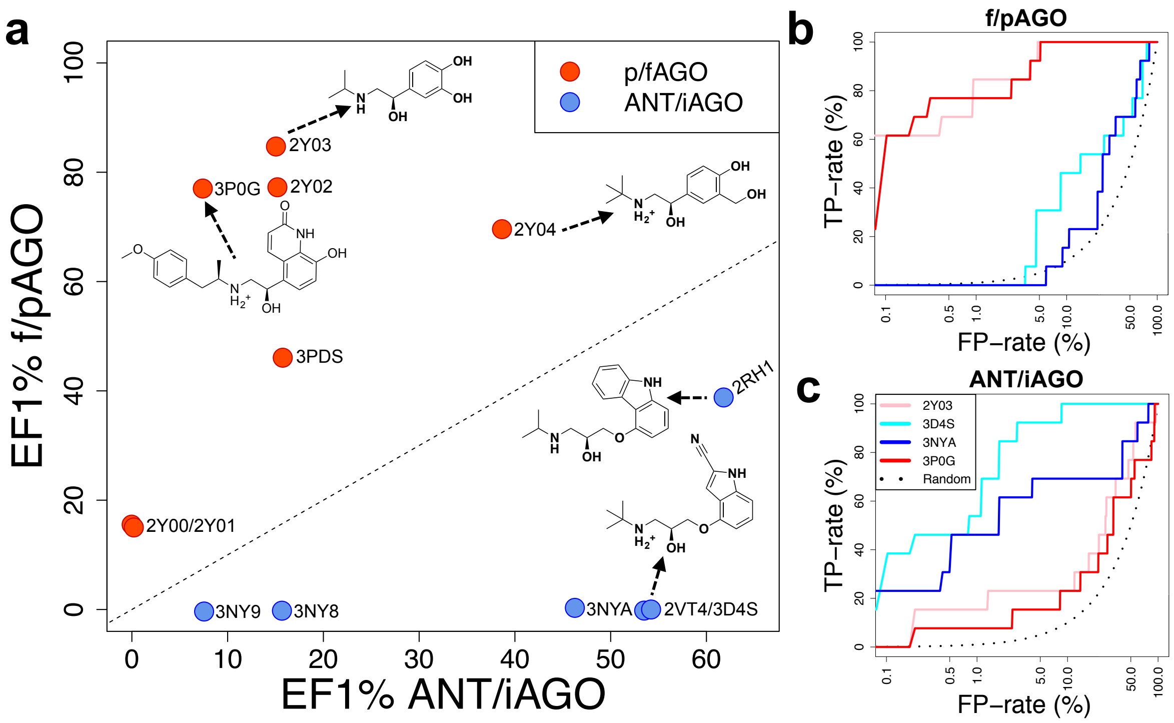

Figure S1. Retrospective validation for the retrieval of agonists over antagonists/inverse agonists over decoys using all ligand-bound β_1R/β_2R crystal structures using IFP-scoring.

For each crystal structure one chain was subjected to docking 20 partial and full agonists (f/pAGO), 21 antagonists (ANT) and inverse agonists (iAGO) and 980 physicochemically similar decoys (Baker, 2005, 2010; de Graaf and Rognan, 2008). The retrieval rate of f/pAGO over decoys and ANT/iAGO over decoys were determined for each of the structures using PLANTS docking and IFP scoring. a) Enrichment factors at a 1% false positive rate for f/pAGO and ANT/iAGO over decoys were determined for all f/p/AGO and ANT/iAGO-bound structures (dots colored red and blue respectively). ROC plots for the retrieval of b) f/pAGO over decoys and c) ANT/iAGO over decoys for two f/pAGO structures (red) and two ANT/iAGO structures (blue) show the selective retrieval of f/pAGO over ANT/iAGO when using the IFP-scoring method. Coloring in subfigure b as shown in the legend of subfigure c.

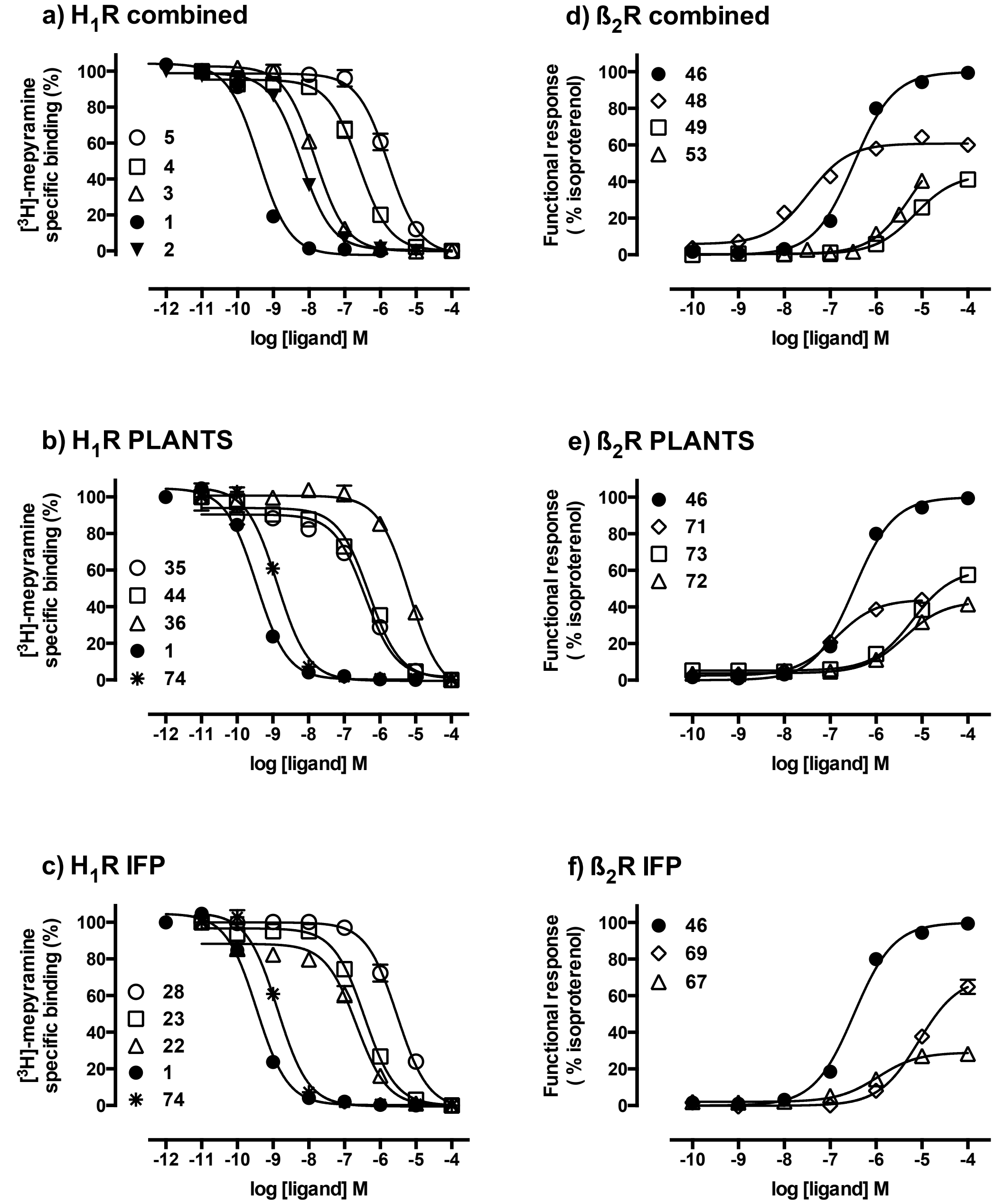

Figure S2. Representative radioligand displacement (H_1R, reference compounds 1, 2, and Mianserin (**74**)) and functional response ($ß_2R$, reference compound **46**) curves of the reference ligands and compounds identified using the combined (a, d), PLANTS (b, e), and IFP (c, f) scoring approaches on H_1R (a-c) and $ß_2R$ (d-f).

References

1 Sato, T. *et al.* Pharmacological Analysis and Structure Determination of 7-Methylcyanopindolol-Bound beta1-Adrenergic Receptor. *Mol. Pharmacol.*, doi:10.1124/mol.115.101030 (2015).

2 Wacker, D. *et al.* Conserved binding mode of human beta2 adrenergic receptor inverse agonists and antagonist revealed by X-ray crystallography. *J. Am. Chem. Soc.* **132**, 11443-11445, doi:10.1021/ja105108q (2010).

3 Christopher, J. A. *et al.* Biophysical fragment screening of the beta1-adrenergic receptor: identification of high affinity arylpiperazine leads using structure-based drug design. *J. Med. Chem.* **56**, 3446-3455, doi:10.1021/jm400140q (2013).

4 Rasmussen, S. G. *et al.* Structure of a nanobody-stabilized active state of the beta(2) adrenoceptor. *Nature* **469**, 175-180, doi:10.1038/nature09648 (2011).

5 Rasmussen, S. G. *et al.* Crystal structure of the beta2 adrenergic receptor-Gs protein complex. *Nature* **477**, 549-555, doi:10.1038/nature10361 (2011).

6 Ring, A. M. *et al.* Adrenaline-activated structure of beta2-adrenoceptor stabilized by an engineered nanobody. *Nature* **502**, 575-579, doi:10.1038/nature12572 (2013).

7 Warne, T., Edwards, P. C., Leslie, A. G. & Tate, C. G. Crystal structures of a stabilized beta1-adrenoceptor bound to the biased agonists bucindolol and carvedilol. *Structure* **20**, 841-849, doi:10.1016/j.str.2012.03.014 (2012).

8 Moukhametzianov, R. *et al.* Two distinct conformations of helix 6 observed in antagonist-bound structures of a beta1-adrenergic receptor. *Proc. Natl. Acad. Sci. U. S. A.* **108**, 8228-8232, doi:10.1073/pnas.1100185108 (2011).

9 Cherezov, V. *et al.* High-resolution crystal structure of an engineered human beta2-adrenergic G protein-coupled receptor. *Science* **318**, 1258-1265, doi:10.1126/science.1150577 (2007).

10 Zou, Y., Weis, W. I. & Kobilka, B. K. N-terminal T4 lysozyme fusion facilitates crystallization of a G protein coupled receptor. *PLoS One* **7**, e46039, doi:10.1371/journal.pone.0046039 (2012).

11 Rasmussen, S. G. *et al.* Crystal structure of the human beta2 adrenergic G-protein-coupled receptor. *Nature* **450**, 383-387, doi:10.1038/nature06325 (2007).

12 Bokoch, M. P. *et al.* Ligand-specific regulation of the extracellular surface of a G-protein-coupled receptor. *Nature* **463**, 108-112, doi:10.1038/nature08650 (2010).

13 Warne, T. *et al.* The structural basis for agonist and partial agonist action on a beta(1)-adrenergic receptor. *Nature* **469**, 241-244, doi:10.1038/nature09746 (2011).

14 Warne, T. *et al.* Structure of a beta1-adrenergic G-protein-coupled receptor. *Nature* **454**, 486-491, doi:10.1038/nature07101 (2008).

15 Miller-Gallacher, J. L. *et al.* The 2.1 A Resolution Structure of Cyanopindolol-Bound beta1-Adrenoceptor Identifies an Intramembrane Na+ Ion that Stabilises the Ligand-Free Receptor. *PLoS One* **9**, e92727, doi:10.1371/journal.pone.0092727 (2014).

16 Weichert, D. *et al.* Covalent agonists for studying G protein-coupled receptor activation. *Proc. Natl. Acad. Sci. U. S. A.* **111**, 10744-10748, doi:10.1073/pnas.1410415111 (2014).

17 Rosenbaum, D. M. *et al*. Structure and function of an irreversible agonist-beta(2) adrenoceptor complex. *Nature* **469**, 236-240, doi:10.1038/nature09665 (2011).

18 Huang, J., Chen, S., Zhang, J. J. & Huang, X. Y. Crystal structure of oligomeric beta1-adrenergic G protein-coupled receptors in ligand-free basal state. *Nat. Struct. Mol. Biol.* **20**, 419-425, doi:10.1038/nsmb.2504 (2013).

19 Hanson, M. A. *et al*. A specific cholesterol binding site is established by the 2.8 A structure of the human beta2-adrenergic receptor. *Structure* **16**, 897-905, doi:10.1016/j.str.2008.05.001 (2008).
